# FGFR1 inhibition in lung squamous cell carcinoma: questions and controversies

**DOI:** 10.1038/cddiscovery.2015.49

**Published:** 2015-11-23

**Authors:** CE Weeden, B Solomon, M-L Asselin-Labat

**Affiliations:** 1 ACRF Stem Cells and Cancer Division, The Walter and Eliza Hall Institute of Medical Research, Melbourne, VIC, Australia; 2 Department of Medical Biology, The University of Melbourne, Parkville, VIC, Australia; 3 Department of Medical Oncology, Peter MacCallum Cancer Centre, East Melbourne, VIC, Australia; 4 Sir Peter MacCallum Department of Oncology, University of Melbourne, Parkville, VIC, Australia

## Abstract

Although the incidence of lung cancer has decreased due to the reduction of tobacco use, lung cancer remains the leading cause of cancer-related death. Lung squamous cell carcinoma represents 30% of lung cancers and only recently have possible drug-targetable mutations been identified in this disease, including fibroblast growth factor receptor 1 (FGFR1) gene amplification and genetic alterations in the phosphoinositide-3 kinase pathway. These discoveries have generated a great interest in the clinic and the initiation of clinical trials using FGFR tyrosine kinase inhibitors to treat FGFR-altered lung cancers. However, preliminary results from these studies have shown that not all patients respond to therapy. Here we review current unresolved questions on the selection of patients for their recruitment in FGFR tyrosine kinase inhibitor trials, how FGFR inhibitors could be combined with other targeted therapies or immunotherapies to improve patient outcome, and how the current preclinical models can help address these questions.

## Facts

- FGFR1 is amplified in 20% of lung squamous cell carcinoma (SqCC).
- Clinical trials using FGFR inhibitors show only partial response to treatment.
- FGFR1 amplification detected by fluorescence *in situ* hybridization (FISH) may not be the right biomarker to predict response to therapy.
- FGFR inhibitors may be combined with other targeted therapy or immunotherapy.

The recent advent of next-generation sequencing technologies has provided us with an in-depth characterization of cancer genomes. In lung cancer, comprehensive high-throughput sequencing data sets are now available for non-small-cell lung cancer (NSCLC) including adenocarcinomas and SqCCs, and small-cell lung cancers (SCLCs).^1–4^ These data sets have not only revealed the genetic landscape of the different lung cancer subtypes, but have enabled tumors to be further classified by their molecular characteristics.

Identification of genetic amplification in fibroblast growth factor receptor 1 (FGFR1) in lung SqCC^5^ has generated immense interest in the use of FGFR inhibitors in the clinic.^6^ However, early results from clinical trials have shown that some, but not all *FGFR1*-amplified tumors are responsive to FGFR-targeted therapy^7,8^ leaving the following questions unresolved:

- Are the right biomarkers being used to predict response to FGFR-targeted therapy?
- Which therapy may be combined with FGFR inhibitors to improve patient outcome?
- How relevant are the current preclinical models to evaluate response to therapy?

## Are the right biomarkers being used to predict response to FGFR-targeted therapy?

**Figure 1:**
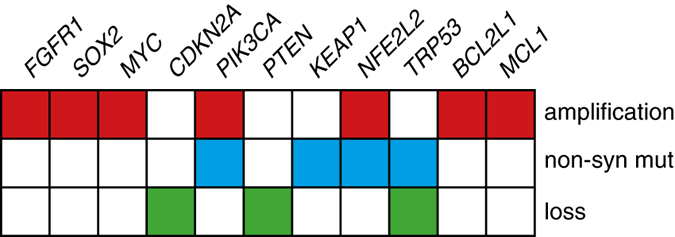
FGFR1 and related genetic alterations present in lung squamous cell carcinoma. Non-syn mut, non-synonymous mutation.

Recent whole-genome sequencing analyses have revealed the complex molecular changes occurring in lung cancer and provide groundwork for the development of personalized medicine in which patients are matched with therapies that best suit the mutation profile of their tumor. There are increasing examples in the clinic with the use of EGFR tyrosine kinase inhibitors (TKIs) in EGFR-mutated lung adenocarcinomas^9,10^ and ALK inhibitors in patients carrying *EML4–ALK* fusion.^11^ The success of personalized medicine relies on the appropriate selection of patients who will respond to treatment. The *FGFR1 8p12* locus is amplified in 20% of lung SqCCs^5^ (Figure 1). However, not all *FGFR1*^*amp*^ cell lines or patient-derived tumors are sensitive to FGFR inhibition,^5,12,13^ suggesting that selection of patients solely based on gene amplification may not prove to be the best predictor of response to therapy. Until now, FGFR1 FISH on tumor biopsies has been the primary strategy utilized to recruit patients to molecularly enriched FGFR TKI trials. Additional biomarkers have been proposed to better predict drug sensitivity, including FGFR1 RNA expression, elevated FGF ligands or activation of downstream signals, but which one to use in a clinical setting remains controversial.

*FGFR1* gene amplification appears to not always correlate with FGFR1 protein or RNA expression in patient tumors, cell lines or patient-derived xenografts (PDXs) models.^12–14^ FGFR1 RNA expression has therefore been proposed as a better predictor of response to FGFR inhibitor therapy.^12^ However, in the study by Wynes *et al.*,^12^ correlation between FGFR RNA expression and sensitivity to FGFR inhibition was performed using ponatinib, a multi-target TKI that potently inhibits multiple tyrosine kinase including BCR-ABL, SRC-related kinases, FLT3, KIT, FGFR1, PDGFR*α*, and VEGFR-2, potentially confounding interpretation of the results.^15^ Using a more specific FGFR inhibitor (PD173074), Dutt *et al.*^16^ showed that *FGFR1*^*amp*^ cell lines that express similar levels of FGFR1 protein have different sensitivity to the inhibitor, indicating that FGFR1 expression may not be sufficient to predict response to FGFR inhibitors.^5^

Autocrine activation of FGFR with endogenous production of ligands may be a predictor of dependency of the cells on FGFR signaling and thus sensitivity to inhibition.^14,17^ Accordingly, elevated phosphorylation of the FGFR substrate FRS2 was associated with increased sensitivity to FGFR inhibitors, suggesting that cells in which FGFR signaling is active may be more sensitive to therapy.^16^ Wynes *et al.*^12^ further proposed that expression of FGF2 and FGF9 in *FGFR1*^*amp*^ lung cancer cell lines was associated with sensitivity to the multi-kinase inhibitor ponatinib. However, activation of FGFR in human tumors may not be cell autonomous with FGF ligands being secreted by cells present in the tumor microenvironment. This proposed correlation between FGF ligands expression and response to FGFR inhibition will have to be further investigated in models of lung cancer that recapitulate the human tumor and its microenvironment, using FGFR-specific inhibitors.

MYC is a regulator of cell proliferation and survival that is overexpressed in cancer but is also involved in cell death.^18^ Malchers *et al.*^17^ found overexpression of MYC in 40% of *FGFR1*^*amp*^ lung SqCCs (Figure 1). They showed that cell lines overexpressing both MYC and FGFR1 were more sensitive to FGFR inhibition compared with cells expressing FGFR1 alone, suggesting that co-expression of MYC may increase sensitivity to FGFR inhibitors. Those results were confirmed in two patients who responded well to FGFR inhibition therapy and had *FGFR1*^*amp*^ tumor overexpressing MYC.^17^ The authors proposed that the pro-apoptotic activity of MYC was necessary to facilitate FGFR inhibitor-induced cell death. However, in *FGFR*^*amp*^/MYC-positive cell lines sensitive to FGFR inhibition, treatment with PD173074, a pan FGFR inhibitor, resulted in downregulation of MYC expression confounding the hypothesis that MYC expression could participate in cell death induced by FGFR inhibition.^17^ These findings would have to be resolved to further determine whether MYC overexpression is associated with FGFR inhibitor sensitivity in FGFR1-amplified lung tumors and if it could be used as a biomarker complementing FGFR1 FISH to predict drug response.

*FGFR1*^*amp*^ has been described predominantly in lung SqCC.^5^ Interestingly, Wynes *et al.*^12^ found that FGFR1-high RNA expression was present in multiple lung cancer types including adenocarcinoma and large-cell carcinoma. Expression of FGFR1 was also observed in SCLC, although correlation with drug sensitivity was not evaluated in these models.^4,19^ Evaluation of the predictive potential of FGFR1 RNA, FGF ligands or MYC expression for response to FGFR inhibitors will need to be further assessed in relevant preclinical models of lung cancer that encompass SqCC as well as adenocarcinoma, large-cell carcinoma and SCLC using FGFR-specific inhibitors, to provide insights into the full spectrum of lung tumors that may respond to FGFR inhibition.

## Which therapy may be combined with FGFR inhibitors to improve patient outcome?

**Figure 2:**
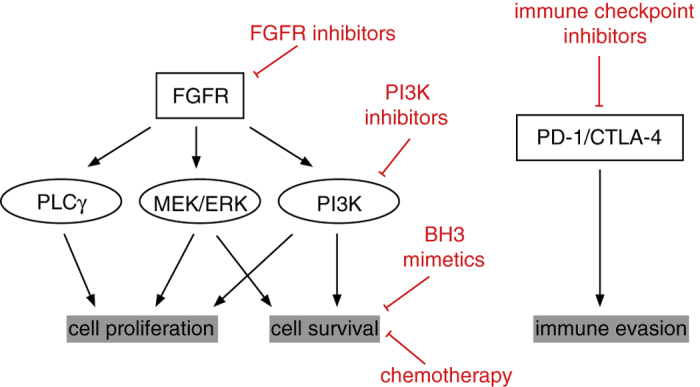
Schematic showing possible combination of FGFR tyrosine kinase inhibitors with other targeted therapy, chemotherapy or immunotherapy.

Intra-tumoral heterogeneity and development of acquired resistance to targeted therapies indicate that combination of multiple therapies are likely to be required for effective long-term control of cancers. To date, clinical trials have shown that single-agent therapy using currently available FGFR inhibitors results in only modest response rates,^7,8^ raising the possibility that the therapeutic response could be enhanced with combination therapy.^13,14,17^ Strategies to improve therapeutic response to FGFR inhibitors could include combination with standard chemotherapy, other targeted therapy or immunotherapy. A recent meta-analysis comparing the activity of EGFR TKI with a combination of EGFR TKI and standard chemotherapy demonstrated no additional benefit of the combination therapy compared with single agent, but a significant increase in toxicity,^20^ indicating that combining TKI with chemotherapy may not prove an appropriate strategy for the patient. Another approach to enhance response to FGFR inhibition, prevent development of acquired resistance and limit the occurrence of adverse events would be to inhibit key components of the pathway downstream of FGFR, including phosphoinositide-3 kinase (PI3K) signaling, or to enhance cell death by combining FGFR inhibitors with pro-apoptotic therapies (Figure 2). Finally, with the recent description of a prolonged survival benefit of immunotherapy in patients with squamous NSCLC demonstrated in a phase-3 trial comparing nivolumab with chemotherapy,^21^ it will be important to investigate whether the combination of immune checkpoint inhibitors with FGFR-targeted therapy may improve patient outcome (Figure 2).

Ligand binding to FGFRs activates three downstream pathways: the Ras-MAP kinase pathway, the PI3K-Akt-mTOR pathway and PLC*γ*-Ca^2+^ pathway to control cell proliferation, survival and differentiation^22^ (Figure 2). Genetic alterations in the PI3 kinase pathway are frequent in lung SqCC with mutations in the catalytic subunit of PI3K encoded by *PIK3CA* present in 5–16%^23^ of lung SqCC, whereas loss of PTEN, a negative regulator of the PI3K pathway, is observed in 15% of tumors^3^ (Figure 1). Twenty-one percent of *PIK3CA-*mutated cancers also present an *FGFR1* amplification.^12^ These overlapping genetic alterations suggest that combining PI3K or mTOR inhibitors with FGFR TKI to increase cell death may improve the therapeutic activity of these agents.^24^ This hypothesis is currently being evaluated in a phase-1b clinical trial with the FGFR inhibitor BGJ398 combined with a PI3K inhibitor (BYL719) in *FGFR1*^*amp*^/*PIK3CA*^*mut*^ cancers^6^ (clinicaltrial.gov; NCT01928459). A study by Faber *et al.*^25^ showed that inhibition of MEK and PI3K resulted in regression of tumor growth in EGFR-mutated lung cancer that had become resistant to EGFR TKI. Similarly, it is possible to envisage that FGFR inhibitor-sensitive tumors may acquire resistance to therapy and that suppression of MEK and the PI3K pathway may provide alternative therapeutics for this subset of SqCC (Figure 2).

Inhibition of growth factor intracellular signaling pathways leads to decreased cell proliferation and/or initiation of apoptosis through induction of the expression of pro-apoptotic BH3-only proteins. Combining TKIs with direct inducers of apoptosis such as BH3 mimetic compounds has been proposed for the treatment of lung cancer.^26^ Cragg *et al.*^27^ showed that induction of BIM by EGFR TKI in EGFR-mutated NSCLC was necessary to induce cell death, and that combination of EGFR TKI with the BH3 mimetic ABT-737 potentiated cell death. Combination of BH3 mimetics with FGFR TKI may similarly improve response to therapy (Figure 2). It will be important to determine which of the anti-apoptotic proteins in the BCL-2 family are the most important survival factors in FGFR1-overexpressing tumors to minimize adverse effects and increase tumor cell death. Analysis of somatic copy-number alterations in multiple cancer types demonstrated amplification of the *MCL1* locus in 10.9% of cancers, with a higher prevalence in lung and breast cancer.^28^ Knockdown of *MCL1* in *MCL1*-amplified cell lines resulted in reduced proliferation and survival, further implying that MCL-1 could be an interesting target in lung cancer. *BCL2L1* that encodes for BCL-XL is also frequently amplified in lung SqCC.^3,28^ Further exploration of the downstream effect of FGFR1 inhibition on the activation of the intrinsic apoptotic pathway will help refine which BH3 mimetic may be the most appropriate for combination therapy with FGFR inhibitors.^29–31^

Immunotherapy has recently shown great promise for the treatment of lung cancer. Initial clinical trials showed that immune checkpoint inhibitors such as the PD1 inhibitor nivolumab prolonged survival from 6 months to 9.2 months in patients with SqCC.^21^ Oncogenes can alter the tumor microenvironment and the nature of immune infiltrates, suggesting that combination immunotherapy and targeted therapy may prove beneficial in oncogene-addicted tumors. In syngenic mouse models of melanoma carrying a *BRAF*^*V600E*^ driver mutation, B-RAF inhibitors have been shown to increase antigen presentation, antigen-specific T-cell recognition and improved T-cell effector function,^32,33^ suggesting that combination of targeted therapy with immunotherapy may improve patient outcome. Hu-Lieskovan *et al.*^34^ indicated that B-RAF inhibitors combined with MEK inhibitors and anti-PD-L1 led to further reduction of tumor growth *in vivo* in a syngeneic mouse model of melanoma. In lung adenocarcinoma, preclinical studies in an EGFR-driven mouse model showed that activation of the EGFR pathway induced PD-L1 expression and recruitment of an increased number of PD1^+^ and Foxp3^+^ regulatory T cells that suppressed effector T-cell function to evade host immune response, suggesting that ablating this immunosuppressive response may augment response to therapy.^35^ Phase-1 clinical trials combining EGFR TKI or ALK inhibitor with anti-PD1 or anti-PD-L1 in EGFR-mutated cancer and ALK-rearranged NSCLC are planned or ongoing (clinicaltrials.gov; NCT02088112; NCT02511184). The question remains to determine whether, similar to what has been described in melanoma or EGFR-mutated lung cancer, FGFR inhibition may result in increased antigen presentation and homing of activated T cells to propose that immunotherapy combined with FGFR inhibitors may enhance therapeutic response (Figure 2). Biomarkers associated with response to checkpoint inhibitors are still poorly understood^36^ and require further study to better select patients who would benefit from such combination therapy. In the case of squamous cell cancer, in contrast to non-squamous NSCLC, mutation load but not PD-L1 expression may be predictive of outcome.^21,37,38^ Ongoing phase-1 and phase-2 studies with checkpoint inhibitors combined with TKI will be critical to determine the safety and efficacy of this therapeutic strategy.

## How relevant are the current preclinical models to evaluate response to therapy?

Preclinical testing of novel therapeutic strategies has largely relied on xenograft models established from tumor cell lines or GEMMs. However, the major caveat of these models is that they do not represent the full heterogeneity of the human disease, and therefore may hamper the development of new therapies.

**Figure 3:**
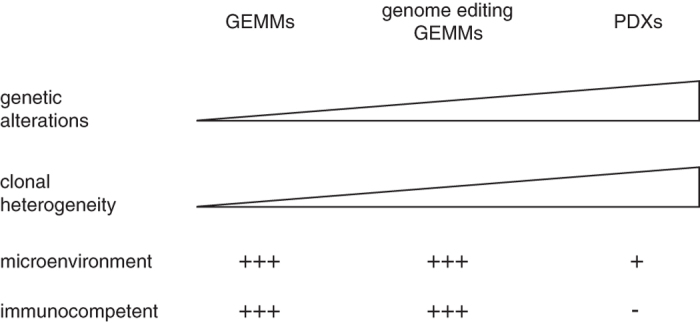
Advantages and limitations of preclinical models of lung cancer.

PDXs are now often used to better represent the intra- and inter-tumor heterogeneity seen in patient samples and have been shown to be a valid preclinical approach to evaluate response to therapy.^39,40^ PDXs are generated from the direct engraftment of resected patient tumor samples into immunocompromised mice. These models have been successfully established for solid tumors, including breast, melanoma, prostate, pancreatic and lung cancers and shown to recapitulate the phenotype, molecular profile and therapeutic response of the patient’s tumors.^13,39,41,42^ As with any model, PDXs have their own pitfalls that need to be considered when investigating response to therapy (Figure 3). PDX models are based on the hypothesis that multiple clones from the patient tumor engraft to reflect intra-tumor heterogeneity. Molecular profiling studies performed on patients’ lung tumor and corresponding PDXs show that the transcriptome and genetic mutations are generally maintained in lung cancer PDXs, indicative of polyclonal engraftment.^43–45^ Analysis of single clones in breast cancer PDXs using deep-genome single-cell sequencing revealed that the clonal diversity in the initial engraftments varies markedly between tumors, with clonal selection occurring at a higher prevalence than polyclonal expansion.^46^ Clonal dynamics occurred during passaging of tumors where limited clonal selection occurred initially.^46^ Similar in-depth studies will need to be performed in lung PDXs to determine whether patterns of clonal selection or polyclonal expansion are observed in these models where the rate of somatic mutations is markedly higher than breast cancer.^47^

Although PDXs may recapitulate the heterogeneity of the patient tumor, they do not maintain the complexity of the tumor microenvironment that includes extracellular matrix (ECM), cancer-associated fibroblasts, and immune and inflammatory cells^48^ (Figure 3). Each tissue has a distinct type of ECM that may transduce different signals to the tumor. This indicates that orthotopic transplantation may be preferable to heterotopic sites such as subcutaneous grafts that are often used for ease of tumor measurement. Eirew *et al.*^46^ showed that the location of the grafts did not affect intra-tumor heterogeneity, but differing signals from the ECM may affect tumor growth, differentiation and metastatic potential.^49^ PDX tumor cells create their own niche by recruiting mouse fibroblasts, however these cells may signal differently to the tumor compared with human cancer-associated fibroblasts. Most importantly, PDXs are transplanted into immunocompromised mice and therefore lack signals from CD8 cytotoxic T cells, tumor-associated macrophages, NK cells or regulatory T cells. Co-engraftment of patient-matched immune cells would most likely be too invasive for the patient to envisage such an option, preventing the use of PDXs models to investigate combination studies of FGFR-targeted inhibitors with immunotherapy. Syngeneic GEMMs would be the most amenable approach to evaluate immunotherapies. However, it is becoming clear that tumors that respond best to immune checkpoint inhibitors, such as melanomas and lung cancers, have a high rate of mutations. This genomic instability promotes the presentation of neo-antigen recognized by immune cells.^37,38^ GEMMs thus far have relied on the introduction of two or three genetic alterations and do not represent the complexity and high rate of mutation present in human tumors, particularly in smoking-associated tumors where the rate of mutation was found to be three times higher than in nonsmokers.^50^ Genome editing using CRISPR/Cas9 may help resolve this issue (Figure 3). Using Cas9 transgenic mice, one could potentially introduce multiple sgRNA to introduce multiple mutations in order to reflect the genomic instability of human lung cancer. Platt *et al.*^51^ demonstrated the feasibility of this approach by intratracheal delivery of an adenovirus expressing a vector encoding three sgRNA targeting *LKB1*, *TRP53*, *K-RAS*, a *K-RAS*^*G12D*^ HDR donor DNA template and cre in *Cas9*^*LSLTg*^ mice. All the animals developed invasive adenocarcinoma in the lung within 2 months of administration of the virus. Such a method has not yet been described to generate mouse models of lung SqCC or FGFR-altered SqCC. A lentiviral approach that permits the integration into the genome of particular constructs^52^ may be more appropriate to enable overexpression of FGFR1 to mimic *FGFR1* amplification, while introducing gene alteration in other *loci* using CRISPR/Cas9.

The development of mouse models using genome-editing approaches is only in the early stage and the studies highlighted above^51,52^ indicate the feasibility of introducing somatic mutations in adult mice rather than working with germline models to understand lung cancer formation.^53^ The high frequency of indels observed with these genome-editing approaches could allow large cohorts of mice to be infected with the viruses and randomized into treatment groups. This would enable the evaluation of drug efficacy in a syngeneic orthotopic environment in order to decipher mechanisms of sensitivity or resistance to therapy.

## Conclusion

The delivery of effective therapy to *FGFR1*-amplified lung SqCC appears to be much more complex than for *EGFR*-mutated or *ALK*-rearranged NSCLC where the genetic alteration highly predicts sensitivity to the associated targeted drug. To date, only modest efficacy has been seen with single-agent FGFR inhibitor therapy. Improved understanding of the pathogenesis of *FGFR1*-amplified tumors, in particular factors that modify sensitivity and mediate resistance to FGFR inhibitors are essential to better select patients and increase the success rate of FGFR inhibitors in the clinic. The intra-tumor heterogeneity found in human malignancies strongly suggests that the use of multiple therapies at once to avoid expansion of a resistant clone may be necessary to effectively treat lung SqCC. Defining the best set of biomarkers that will predict response to particular combination therapies is instrumental for the development of better personalized medicine with the ultimate goal of improving outcomes for patients with lung SqCC.

## Abbreviations

ECM: extracellular matrix
FGFR1: fibroblast growth factor receptor 1
FISH: fluorescence *in situ* hybridization
GEMM: genetically engineered mouse model
NSCLC: non-small-cell lung cancer
PDX: patient-derived xenograft
PI3K: phosphoinositide-3 kinase
SqCC: squamous cell carcinoma
TKI: tyrosine kinase inhibitor.
